# Competition-cooperation relationship networks characterize the competition and cooperation between proteins

**DOI:** 10.1038/srep11619

**Published:** 2015-06-25

**Authors:** Hong Li, Yuan Zhou, Ziding Zhang

**Affiliations:** 1 State Key Laboratory of Agrobiotechnology, College of Biological Sciences, China Agricultural University, Beijing 100193, China

## Abstract

By analyzing protein-protein interaction (PPI) networks, one can find that a protein may have multiple binding partners. However, it is difficult to determine whether the interactions with these partners occur simultaneously from binary PPIs alone. Here, we construct the yeast and human competition-cooperation relationship networks (CCRNs) based on protein structural interactomes to clearly exhibit the relationship (competition or cooperation) between two partners of the same protein. If two partners compete for the same interaction interface, they would be connected by a competitive edge; otherwise, they would be connected by a cooperative edge. The properties of three kinds of hubs (i.e., competitive, modest, and cooperative hubs) are analyzed in the CCRNs. Our results show that competitive hubs have higher clustering coefficients and form clusters in the human CCRN, but these tendencies are not observed in the yeast CCRN. We find that the human-specific proteins contribute significantly to these differences. Subsequently, we conduct a series of computational experiments to investigate the regulatory mechanisms that avoid competition between proteins. Our comprehensive analyses reveal that for most yeast and human protein competitors, transcriptional regulation plays an important role. Moreover, the human-specific proteins have a particular preference for other regulatory mechanisms, such as alternative splicing.

Protein-protein interaction (PPI) networks lend a systematic perspective to the studies of cellular functions and biological processes. Since the first yeast and human PPI networks were established in 2000 and 2005 respectively^1^,^2^, the computational study of PPI networks has become an important research topic in the field of systems biology. With the development of high-throughput experimental methods and computational approaches^3^,^4^, a large number of interactions have been detected. In the past decade, many studies have reported the characterization of PPI network properties, including the scale-free property^5^. Most proteins have low connectivity, while a small number of proteins, called hubs, have more protein interaction partners in the networks^6^,^7^. Moreover, hubs can be further divided into two categories: ‘date’ hubs and ‘party’ hubs, or single-interface hubs and multi-interface hubs. Both ‘date’ hubs and ‘party’ hubs play important roles in the modular organization of PPI networks. ‘Date’ hubs tend to link different modules and have low expression correlations with their partners, while ‘party’ hubs tend to link different members in one module and have gene expression patterns highly correlated with those of their partners^7^,^8^. Single-interface hubs, just as the name implies, have at most two interaction interfaces that are shared by multiple partners. In contrast, multi-interface hubs contain more than two interaction interfaces and can interact with different partners simultaneously. Generally, ‘date’ hubs tend to be single-interface hubs and ‘party’ hubs tend to be multi-interface hubs^9^.

PPI networks have been integrated with various data in order to provide a systematic understanding of diverse biological processes^10^,^11^. Among them, protein structure data are of particular importance. Although sometimes experimentally derived protein-protein complex structures are not available, homology modeling can be used to produce the models of protein-protein complexes based on the solved structures of protein complex templates^12^. Therefore, it is possible to analyze the interaction interfaces between proteins at atomic resolution. It is worth mentioning that the proteins interacting with many partners can have either multiple interfaces or just one interface. Some of these interfaces are shared by different partners, resulting in mutually exclusive bindings; other interfaces are used by only one partner such that the interactions with different partners occur simultaneously. Thus, to judge whether two partners can interact with the common protein simultaneously, the key is to know whether they share an interaction interface.

Gene duplication promotes evolution and can increase the competition between proteins that bind the same interaction interface^13^,^14^. The analysis of competition or cooperation between two proteins contributes to the understanding of cellular machinery. However, traditional PPI networks cannot reflect these competitive and cooperative relationships. An effective solution is to construct a new type of network. For example, by dividing proteins into interfaces, Johnson and Hummer have constructed an interface-interaction network that allows the visualization of protein interactions at the interface level. That is to say, in this network, nodes represent interfaces instead of proteins, and edges represent interactions between interfaces. If a node connects more than one edge, the corresponding interactions cannot happen simultaneously^15^. The interface-interaction network provides detailed information regarding individual interfaces, which is important for protein engineering and drug design. However, it does not provide a straightforward way to analyze how extensive competition is regulated *in vivo*, because most of the related data (e.g., the gene expression data and the alternative splicing information) do not describe interfaces but rather proteins or genes. As a result, defining interfaces as nodes in the network may hamper further systematic analyses.

Here, we combine PPI networks with protein structure data to construct the yeast and human competition-cooperation relationship networks (CCRNs; Fig. 1). Each node corresponds to a protein, and each edge describes a relationship (i.e., competition or cooperation) between two proteins. We analyze competitive, modest and cooperative hubs in these CCRNs. The results show that competitive hubs have higher clustering coefficients and form multiple clusters in the human CCRN but not in the yeast CCRN. We ascribe these differences mainly to the emergence of human-specific proteins. Indeed, the human basic CCRN, in which the human-specific proteins are omitted, exhibits topological features much more similar to those of the yeast CCRN than those of the human CCRN. Interestingly, we also find that the human-specific proteins are regulated in a distinct fashion in which intensive competitive interactions are avoided.

## Results

### Competition-cooperation relationship networks

**Figure 1:**
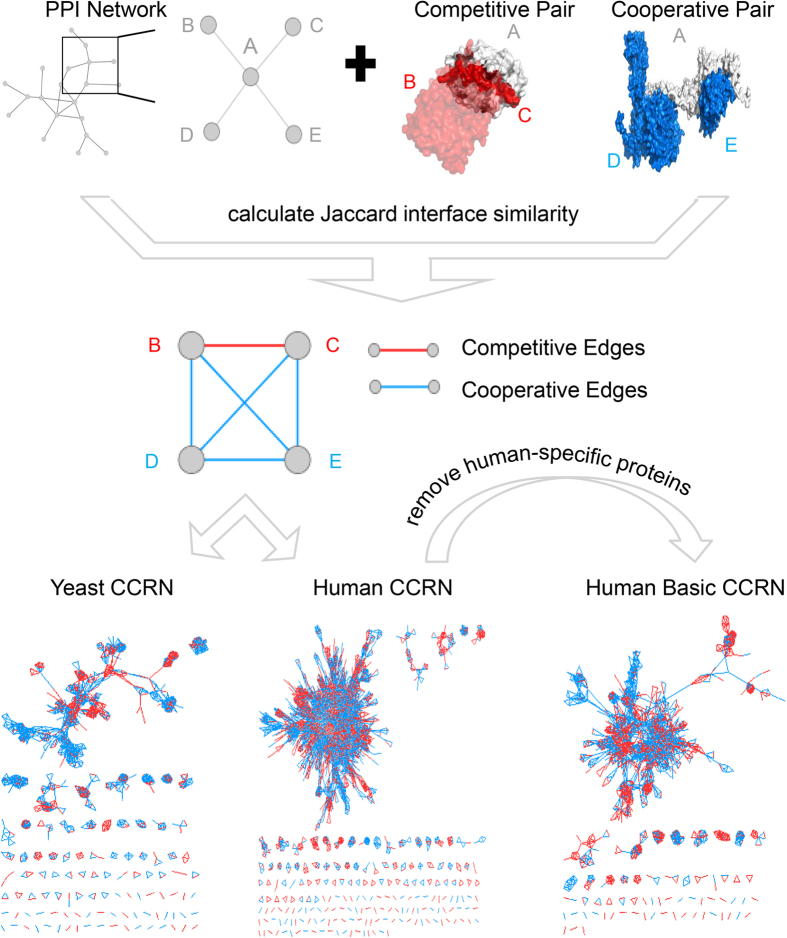
Construction of the CCRNs. In this figure, protein B, C, D and E all interact with the common protein A. There are four interaction interfaces on protein A: interface BA, CA, DA and EA. By comparing any two interfaces (BA-CA, BA-DA, BA-EA, CA-DA, CA-EA, DA-EA), we could obtain six values of Jaccard interface similarity for the interfaces. Here, the Jaccard interface similarity between interface BA and CA is larger than 0.1; protein B and C are therefore defined as a competitive pair, connected by a competitive edge (red). In contrast, the Jaccard interface similarity between DA and EA is less than 0.1; protein D and E are therefore defined as a cooperative pair, connected by a cooperative edge (blue). While protein B (or C) and D (or E) bind different interfaces of A. Therefore, the relationship between B (or C) and D (or E) is also cooperative. By such procedure, the yeast and human PPI networks were combined with 3D structural data to construct the yeast and human CCRNs. The human basic CCRN was constructed by removing the human-specific proteins from the human CCRN.

To depict the competitive and cooperative relationships between proteins more intuitively, the yeast and human CCRNs were constructed based on protein structural interactomes. The CCRNs are undirected and bicolor networks, in which each edge describes a competitive or cooperative relationship between two proteins. If two proteins interact with a common protein through the same interaction interface, they are defined as a competitive pair connected by a competitive edge. In contrast, two proteins are defined as a cooperative pair connected by a cooperative edge, if they bind the same protein at different interaction interfaces. As a result, we obtain a yeast CCRN containing 823 proteins connected by 1,244 competitive edges and 1,964 cooperative edges. We also constructed a human CCRN containing 2,897 proteins connected by 10,463 competitive edges and 11,048 cooperative edges (Fig. 1; the raw network data of the CCRNs and the full list of their edges are available at http://systbio.cau.edu.cn/CCRN/). We performed the network topology analyses for both yeast and human CCRNs. We find that both CCRNs fit a power law distribution, like other biological networks [*P*(*k*) = 0.532 × *k*^−1.403^ for the yeast CCRN and *P*(*k*) = 0.733 × *k*^−1.529^ for the human CCRN, where the degree distribution *P*(*k*) gives the proportion of nodes with degree *k*]. Compared with PPI networks, however, some different network properties also exist in these two CCRNs, including a higher average degree and a smaller number of connected components (Supplementary Table S1).

### Competitive hubs have higher clustering coefficients

We notice that some proteins prefer to use the same interaction interfaces on the common proteins, whereas some other proteins favor to interact with the common proteins by using different interaction interfaces. What is the difference between the two types of proteins in the CCRNs? To answer this question, we first defined the proteins whose degrees ranked in the top 20% as hubs in a CCRN. Then, we classified the hubs as competitive hubs, modest hubs or cooperative hubs according to their percentage of connected competitive edges. As the names imply, competitive hubs are the proteins that tend to compete for the same interaction interfaces, while cooperative hubs are the proteins that tend to use the different interaction interfaces on the common proteins. There are 44 competitive hubs, 31 modest hubs and 89 cooperative hubs in the yeast CCRN, while the corresponding hub numbers in the human CCRN are 222, 166 and 191, respectively.

**Figure 2:**
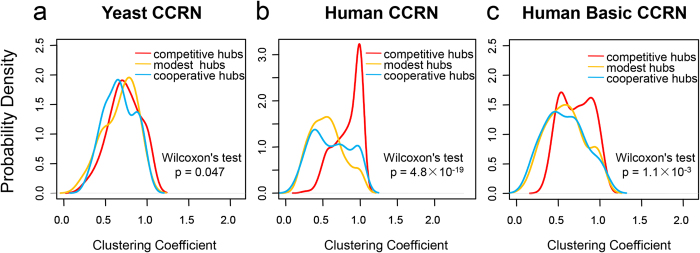
Comparison of clustering coefficient distributions. The difference in clustering coefficient distributions between competitive hubs and cooperative hubs is estimated using one-tailed Wilcoxon’s test.

Intuitively, the partners of competitive hubs tend to be tightly linked with each other in the human CCRN (Supplementary Fig. S1). To quantify this property, we calculated the network topology parameter clustering coefficient for each hub. The higher clustering coefficient of a hub is, the more tightly its partners link with each other. The maximum clustering coefficients can reach to 1.0 for competitive and cooperative hubs in both yeast and human CCRNs. In the yeast CCRN, the minimum clustering coefficients for competitive and cooperative hubs are 0.258 and 0.276, respectively; while in the human CCRN, the corresponding values are 0.257 and 0.114. We further compared the distributions of clustering coefficients between competitive hubs and cooperative hubs in the yeast and human CCRNs. Our results show that competitive and cooperative hubs have similar distributions of clustering coefficients in the yeast CCRN (Fig. 2a), although the average clustering coefficient of competitive hubs (0.727) is slightly higher than the average clustering coefficient of cooperative hubs (0.668). However, in the human CCRN, there is a significant difference between competitive and cooperative hubs (the significance threshold is set as p-value < 0.01 in this study) in the distributions of clustering coefficients (one-tailed Wilcoxon’s test, p-value = 4.8 × 10^−19^). Moreover, a sharp peak close to 1 can be observed in the distribution for competitive hubs (Fig. 2b), leaving no doubt that the average clustering coefficient of competitive hubs (0.835) is higher, even 1.4 times as high, than that of cooperative hubs (0.609).

The higher clustering coefficients observed for competitive hubs may indicate that competitive edges are more likely to form clusters in the human CCRN. To further validate this, we performed one additional analysis. By only considering competitive edges for competitive hubs and cooperative edges for cooperative hubs, we re-calculated their clustering coefficients and compared the distributions of clustering coefficients between competitive hubs and cooperative hubs. We find that competitive hubs have even more significantly higher clustering coefficients than cooperative hubs (one-tailed Wilcoxon’s test, p-value = 4.8 × 10^−57^; Supplementary Fig. S2). This result confirms that competitive hubs tend to locate in a cluster of proteins densely linked by competitive edges, or in other words, competitive edges tend to aggregate in the human CCRN.

The apparent divergence between the yeast and human CCRNs spurs our further investigation. We find that some protein domains only exist in the human CCRN, according to the Pfam domain annotations^16^. We referred these domains as the human-specific domains and defined the proteins containing the human-specific domains as the human-specific proteins (the full list of human-specific proteins is available at http://systbio.cau.edu.cn/CCRN/). All other proteins in the human CCRN were defined as the human non-specific proteins. By removing the human-specific proteins from the human CCRN, the human basic CCRN was constructed, which consists of 995 nodes and 4,186 edges (including 2,268 competitive edges and 1,918 cooperative edges; the raw data of human basic CCRN are available at http://systbio.cau.edu.cn/CCRN/). To determine if the human-specific proteins cause the divergence between the yeast and human CCRNs, we further analyzed the distributions of clustering coefficients for the three types of hubs in the human basic CCRN. By applying the same criteria, we classified 79 hubs as competitive hubs, 83 hubs as modest hubs and 37 hubs as cooperative hubs in the human basic CCRN. Indeed, as what we have speculated, competitive hubs and cooperative hubs just show little difference in the distributions of clustering coefficients in the human basic CCRN, and this difference is much marginal compared with the difference observed in the human CCRN [one-tailed Wilcoxon’s test, p-value = 1.1 × 10^−3^ for the human basic CCRN (Fig. 2c) and p-value = 4.8 × 10^−19^ for the human CCRN (Fig. 2b)]. This result implies that the human-specific proteins do contribute to the divergence between the yeast and human CCRNs.

### Competitive hubs tend to locate in the network modules

We investigated the organization of different kinds of hubs with respect to the network’s modular structure. For each CCRN, the largest connected component was divided into modules using the Markov cluster algorithm (http://micans.org/mcl/) to calculate the participation coefficients of their hubs. The participation coefficient of a hub is close to 1 when its links are equally distributed among all of the modules, and is equal to 0 when its links are all included within its own module^17^. In other words, a lower participation coefficient for a hub denotes that the hub is more likely to be intra-module and has a more compact connection within its module. As expected, competitive hubs and cooperative hubs show very similar distributions of participation coefficients in the yeast CCRN (Supplementary Fig. S3). At the same time, both distributions present peaks of approximately 0, indicating that most of the hubs are intra-module hubs. However, in the human CCRN, two distributions differ significantly as competitive hubs have lower participation coefficients compared to cooperative hubs (one-tailed Wilcoxon’s test, p-value = 3.4 × 10^−16^; Supplementary Fig. S3). For the human basic CCRN, similar to the situation when analyzing the clustering coefficient, there is much smaller difference in the participation coefficient between competitive hubs and cooperative hubs (one-tailed Wilcoxon’s test, p-value = 9.8 × 10^−5^; Supplementary Fig. S3), in comparison with the human CCRN.

**Figure 3:**
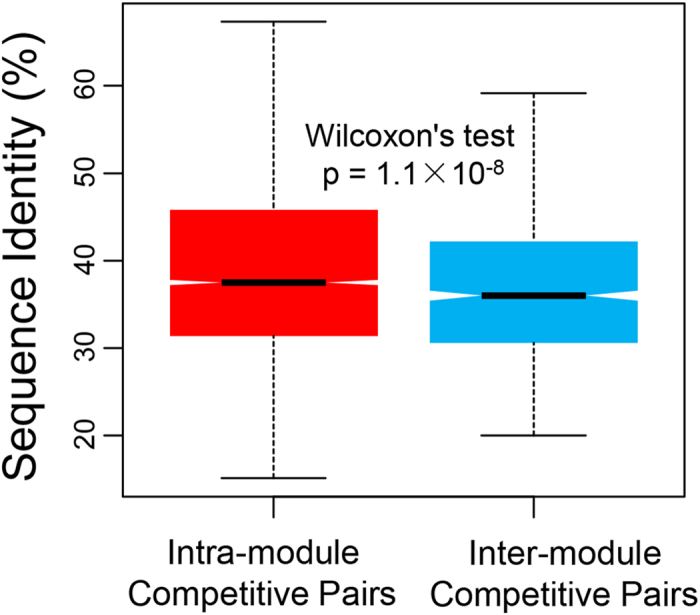
Comparison of sequence identity distributions. The sequence identities between intra-module competitive pairs and those between inter-module competitive pairs are compared. The difference in sequence identity distributions is estimated using one-tailed Wilcoxon’s test. The black line indicates the median. The range of the box is from the first quartile to the third quartile.

The lower participation coefficients of competitive hubs in the human CCRN imply that these hubs tend to locate within the network modules (clusters). In order to give a more clear view, competitive hubs were highlighted using red color on the largest connected component of human CCRN (Supplementary Fig. S1; see also http://systbio.cau.edu.cn/CCRN/ for more detailed data). Intuitively, competitive hubs tend to form multiple clusters in the network. To explain this phenomenon, we further analyzed the contribution of paralogous proteins. Firstly, all competitive pairs (i.e., the protein pairs connected by competitive edges) were clustered into modules by the Markov cluster algorithm, and the modules containing at least one competitive hub were defined as the hub-associated modules. The sequence identities between intra-module competitive pairs and those between inter-module competitive pairs were compared subsequently. The intra-module competitive pairs are those appear in the same hub-associated module, while a competitive pair is deemed inter-module if one of the proteins belongs to a hub-associated module and the other one appears in a different module. The results show that the intra-module competitive pairs have higher sequence identities than the inter-module competitive pairs (one-tailed Wilcoxon’s test, p-value = 1.1 × 10^−8^; Fig. 3). This implies that the intensive competition between proteins, which is demonstrated by the clusters surrounding competitive hubs, may be partly caused by the emergence of paralogous proteins^14^.

### Cooperative hubs are more likely to be essential

We probed the potential roles which different hubs might play in cells and focused on the comparison of protein essentiality for competitive and cooperative hubs. We find that the essential proteins encoded by the essential genes for an organism’s survival are more significantly enriched in cooperative hubs compared with competitive hubs, in both yeast and human CCRNs (one-tailed Fisher’s exact test, p-value = 3.6 × 10^−4^ for the yeast CCRN and p-value = 4.4 × 10^−5^ for the human CCRN; Table 1). This is mainly due to the fact that essentiality is a property of the protein complex^13^; and intuitively, cooperative hubs are more likely to appear in the protein complexes. In addition, we have identified the hubs involved in disease according to their UniProt annotations^18^. The analysis shows that cooperative hubs are more likely to be associated with disease than competitive hubs (one-tailed Fisher’s exact test, p-value = 3.7 × 10^−4^). However, we find that cooperative hubs are neither likely to be essential nor to have a strong correlation with disease in the human basic CCRN, partly due to the limited number of cooperative hubs in the human basic CCRN (Table 1).

### Spatiotemporal divergence of gene expression reduces the competition between proteins

**Figure 4:**
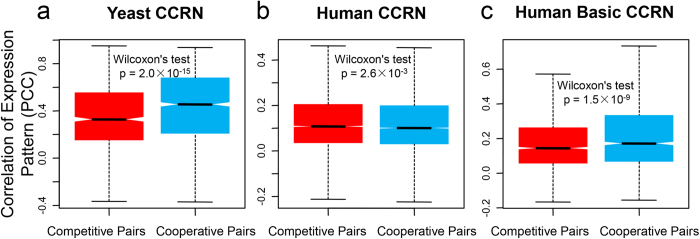
Comparison of PCC distributions. The correlation of gene expression patterns for a pair of proteins is quantified by PCC. The p-value is estimated from one-tailed Wilcoxon’s test. The black line indicates the median. The range of the box is from the first quartile to the third quartile.

Since competition for an interaction interface between proteins appears to be a common phenomenon in the organisms, it is important to ask how cellular machinery avoids this kind of competition. It has been suggested in the yeast PPI network that ‘party’ hubs tend to co-express with their partners, whereas there is no obvious co-expression patterns between ‘date’ hubs and their partners; i.e., ‘date’ hubs interact with different partners at different time and locations^7^,^19^. We supposed that the competitive pairs in the CCRNs would also have different gene expression patterns in order to avoid competition. To test this hypothesis, we employed the Pearson correlation coefficient (PCC) to quantify the correlation of gene expression patterns between proteins and compared the PCC distributions for all of the competitive pairs (i.e., the protein pairs connected by competitive edges) and the cooperative pairs (i.e., the protein pairs connected by cooperative edges) in the yeast and human CCRNs. In accordance with our conjecture, there are much lower PCCs overall for competitive pairs than those for cooperative pairs in the yeast CCRN (one-tailed Wilcoxon’s test, p-value = 2.0 × 10^−15^). The median PCC of competitive pairs (0.329) is also much lower than that of cooperative pairs (0.455; Fig 4a). This result reveals that transcriptional regulation could be a common strategy in yeast for inducing the spatiotemporal divergence of gene expression among competitive protein pairs to relieve their interaction competition.

As described above, our results have showed that competitive hubs tend to locate in compact clusters in the human CCRN (Fig. 2b); namely, more intensive competition exists in human. We assumed that the significant divergences of gene expression patterns between competitive pairs would also be observed in the human CCRN to mediate this intense competition. However, we are surprised to find that the correlations between competitive pairs (median PCC = 0.108) are even marginally higher relative to cooperative pairs (median PCC = 0.101) (one-tailed Wilcoxon’s test, p-value = 2.6 × 10^−3^; Fig. 4b). To explain this discrepancy between the yeast and human CCRNs, we compared the PCC distributions between competitive and cooperative pairs in the human basic CCRN. In the human basic CCRN, we find lower PCCs for competitive pairs, mimicking the result from the yeast CCRN (one-tailed Wilcoxon’s test, p-value = 1.5 × 10^−9^; Fig. 4c). This indicates that transcriptional regulation is a common way to evade competition in human, with the exception of the human-specific proteins. The human-specific proteins would employ alternative mechanisms, e.g., alternative splicing and domain co-occurrence, to regulate the competition, as shown below.

### Alternative splicing reduces the competition involving the human-specific proteins

Alternative splicing is an intermediate regulatory process between transcription and translation^20^,^21^. It is known that alternative splicing increases the diversity of proteome by producing a variety of isoforms^22^. When a gene abundance is constant, the more isoforms the gene has, the less abundant a given isoform will be. Moreover, the noisy splicing that drives the diversity of isoform also decreases the protein abundance^23^. Finally, if the domains involved in the competition are removed by alternative splicing, the competition will be eliminated naturally. To determine if alternative splicing is a way to avoid the competition in human, we extracted the isoform information for the human-specific proteins and the human non-specific proteins from the UniProt database^18^. By definition, the proteins containing at least two isoforms are regulated by alternative splicing. Our results show that the human-specific proteins are enriched among the set of proteins containing two or more isoforms (one-tailed Fisher’s exact test, p-value = 7.2 × 10^−3^; Table 2), indicating that they tend to be regulated by alternative splicing. After the exclusion of the proteins that are not alternatively spliced, we further compared the isoform numbers of the human-specific proteins and the human non-specific proteins. We find that the human-specific proteins have more isoforms on the whole (one-tailed Wilcoxon’s test, p-value = 1.3 × 10^−4^; Supplementary Fig. S4). Moreover, we also find that the interaction domains of the human-specific proteins have a higher fraction of alternatively spliced residues (one-tailed Wilcoxon’s test, p-value = 3.2 × 10^−6^) and a higher fraction of natural variant residues (one-tailed Wilcoxon’s test, p-value = 1.5 × 10^−10^).

It is also possible that the human-specific proteins employ other mechanisms to evade competition. We compared the number of domain types between the human-specific proteins and the human non-specific proteins. The results show that there are more domain types in the human-specific proteins on the whole (one-tailed Wilcoxon’s test, p-value = 6.7 × 10^−86^) and that the human-specific proteins are especially enriched among the proteins with multiple (>2) domain types (Supplementary Fig. S5). Therefore, it is plausible that the appearance of multiple domains would establish another layer of competition regulation for the human-specific proteins via domain interplay.

### Case studies

**Figure 5:**
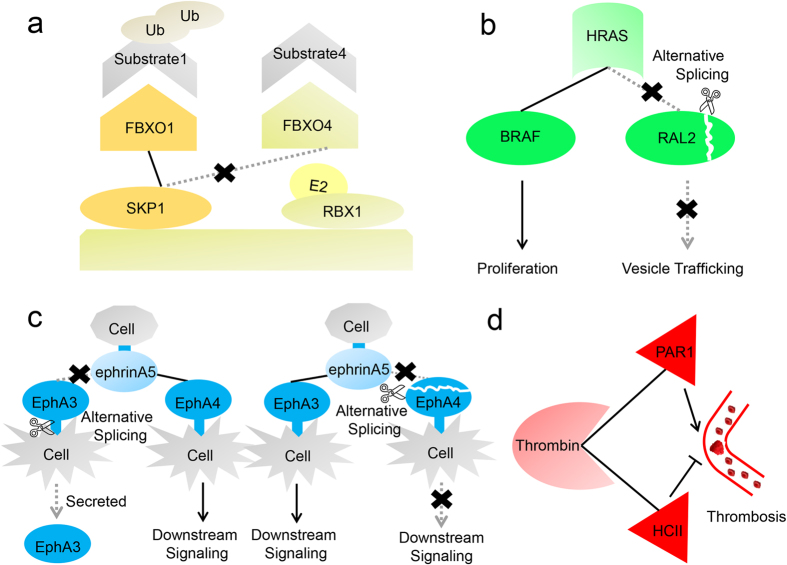
Case studies of competitive relationships. (**a**) FBXO1 and FBXO4 compete for the same interaction interface on SKP1. Here, FBXO1 binds SKP1 to mediate the degradation of Substrate 1, while the gene expression of FBXO4 is inhibited to avoid potential competition. (**b**) BRAF interacts with HRAS to affect proliferation. Likewise, RAL2 also interacts with HRAS to trigger vesicle trafficking. However, once RAL2’s C-terminal interaction domain is removed by alternative splicing, the competitive interaction which it is engaged in will be eliminated. (**c**) EphA3 and EphA4 competitively interact with the ligand ephrin-A5 residing on an adjacent cell to mediate contact-dependent cell-cell communication. EphA3 is secreted if its transmembrane and cytoplasmic domains are removed by alternative splicing, while the ephrin-binding domain of EphA4 is also under the regulation of alternative splicing, which can relieve the competition between EphA3 and EphA4. (**d**) Thrombin promotes thrombosis when interacting with PAR1. In contrast, HCII can occupy the interaction interface shared with PAR1 to exert its anti-thrombin action.

Our analyses have revealed that different gene expression patterns and alternative splicing are potential mechanisms for reducing competition between proteins in cells, which are further exemplified through the following case studies (Fig 5). Figure 5a depicts the competition between FBXO1 and FBXO4. The F-box family has 69 members in human, including FBXO1 and FBXO4. As alternative substrate adaptors, FBXO1 and FBXO4 bind SKP1 competitively and each of them can form a transient SCP complex with CUL1 and RBX1 to mediate the degradation of different substrate proteins. Our results show that the PCC of gene expression patterns between FBXO1 and FBXO4 is low (0.083), which is in line with these two adaptors’ temporary activation via conditional gene expression^24^. That is to say, usually only one of FBXO1 and FBXO4 can be expressed at a sufficient level to exert its adaptor function by interacting with SKP1 and the corresponding substrates.

Figure 5b depicts the competition between BRAF and RAL2. HRAS protein is a molecular switch that activates different pathways when coupled to different downstream effectors. Both BRAF and RAL2 are effectors of HRAS and compete for a shared interaction interface on HRAS. It has been reported that BRAF activates the MEK–ERK kinase cascade to regulate proliferation^25^. Recently, four additional isoforms of BRAF, produced by alternative splicing, have been discovered. The C-terminals of these isoforms are truncated, which affects the overall cellular BRAF activity^26^. RAL2 acts on another pathway to regulate vesicle trafficking^25^. Similar to BRAF, two isoforms of RAL2 are produced. One isoform lacks the RA domain (which is the interaction domain that binds HRAS), resulting in that RAL2 cannot interact with HRAS. These alternative splicing events greatly reduce the number of proteins capable to bind HRAS.

The regulation of alternative splicing is further exemplified by two EphA receptors (EphA3 and EphA4) (Fig. 5c). EphA3 and EphA4 competitively interact with the ligand ephrin-A5 residing on an adjacent cell to mediate contact-dependent cell-cell communication. Both EphA3 and EphA4 are constituted by an extracellular ephrin ligand-binding domain, a transmembrane segment and a cytoplasmic region that contains the kinase domain^27^. Due to the lack of the transmembrane segment and the cytoplasmic kinase domain, one isoform of EphA3 is secreted instead of being embedded in the plasma membrane and cannot perform its function properly. Moreover, the ephrin ligand-binding domain is truncated in one isoform of EphA4, prohibiting the interaction between EphA4 and ephrin-A5. Therefore, the competition between EphA3 and EphA4 would be relieved by these alternative splicing events.

Finally, it is worth mentioning that the competition should not be treated as a disorder but as a sophisticated way to organize the protein interactions. For example, PAR1 and HCII elicit opposing functions upon interacting with thrombin. More importantly, they bind the same interaction interface (Fig. 5d). Thrombin can act on PAR1 to accelerate platelet aggregation in order to stop bleeding. However, the pathological interaction between thrombin and PAR1 can also stimulate thrombosis and even induce the formation of atherosclerotic lesions^28^. It is therefore important to inhibit the activation of thrombin at the appropriate time. HCII occupies the common interaction interface to exert its anti-thrombin action^29^,^30^,^31^,^32^. This inhibition is apparently vital to avoid pathological thrombus formation.

### Verification of the results using alternative criteria

We have noted that several factors may substantially influence our major conclusions, including a) the definition of interface residues; b) the threshold for identifying hub proteins; and c) the scope of human-specific proteins. To test the robustness of our conclusions, we performed the related analyses again with alternative criteria for these three factors. In general, our conclusions still hold when the alternative criteria are applied. The detailed results are reported as follows.

To build a CCRN, the first step is the definition of interface residues. In the above analyses, the C_β_ atom (C_α_ for glycine) distance cutoff of 7.5 Å was used to identify interface residues^33^. However, there are many alternative definitions of interacting residues between interfaces. To test the reliability of our conclusions, we took one popular alternative definition, where a residue is defined as an interface residue if any of its heavy atoms is no more than 4 Å away from the heavy atoms of the protein interaction partner^34^. Most of the analyses in this study have been revisited accordingly. The results show that all our conclusions remain unchanged, including: a) competitive hubs have higher clustering coefficients in the human CCRN and the human basic CCRN, but not in the yeast CCRN (Supplementary Fig. S6); b) cooperative hubs have higher participation coefficients in the human CCRN and the human basic CCRN but not in the yeast CCRN (Supplementary Fig. S7); c) essential proteins are enriched in cooperative hubs instead of competitive hubs in both yeast and human CCRNs (Supplementary Table S2); d) competitive pairs have higher PCCs in the human CCRN but lower PCCs in the yeast CCRN or the human basic CCRN (Supplementary Fig. S8).

Moreover, to further check the robustness of our conclusions about hub proteins, we applied different definitions of hub proteins and repeated the corresponding analyses. In each of the repeated analyses, the proteins ranked in the top 10%, 15%, 25% or 30% of degree distribution were defined as hubs, respectively. We find that all of the results are consistent when different thresholds are applied. a) For clustering coefficient, there is no significant difference between competitive and cooperative hubs in the yeast CCRN. However, in the human CCRN and the human basic CCRN, competitive hubs have higher clustering coefficients (Supplementary Fig. S9). b) For participation coefficient, there is also no significant difference between competitive and cooperative hubs in the yeast CCRN. However, in the human CCRN and the human basic CCRN, competitive hubs have lower participation coefficients (Supplementary Fig. S10). c) In comparison with competitive hubs, cooperative hubs are more likely to be essential in the yeast and human CCRNs (Supplementary Table S3).

Finally, in the aforementioned analyses where Pfam domain information has been employed to identify the human-specific proteins, we have found that the human-specific proteins play an important role in the divergence between the yeast and human CCRNs. However, the coverage of current domain annotation database may influence our results. To address this issue, as the alternative to the Pfam domain information, high-coverage protein family information from the PANTHER database^35^ was employed to identify the human-specific proteins. We extracted the PANTHER families only existing in the human CCRN and defined the proteins belonging to these families as the human-specific proteins. The rest of the proteins in the human CCRN were classified as the human non-specific proteins. Furthermore, a new human basic CCRN was constructed by removing these human-specific proteins from the human CCRN. We find that all of the new results are in line with the previous results based on the Pfam domain information. a) Compared with cooperative hubs, competitive hubs have higher clustering coefficients in the new human basic CCRN, but the difference is much smaller than that in the human CCRN (Supplementary Fig. S11); b) The participation coefficients for competitive hubs are lower than those for cooperative hubs, while the difference between them is also much smaller than that in the human CCRN (Supplementary Fig. S11); c) Essential proteins are still not enriched in the cooperative hubs of the new human basic CCRN (Supplementary Table S4); d) Competitive pairs have lower gene expression correlations (PCCs) than cooperative pairs in the new human basic CCRN (Supplementary Fig. S11); e) The human-specific proteins are enriched among the set of proteins containing two or more isoform entries (Supplementary Table S5) and have more isoforms after the exclusion of the proteins with one isoform (Supplementary Fig. S11).

### Analyses about the role of yeast-specific proteins

**Figure 6:**
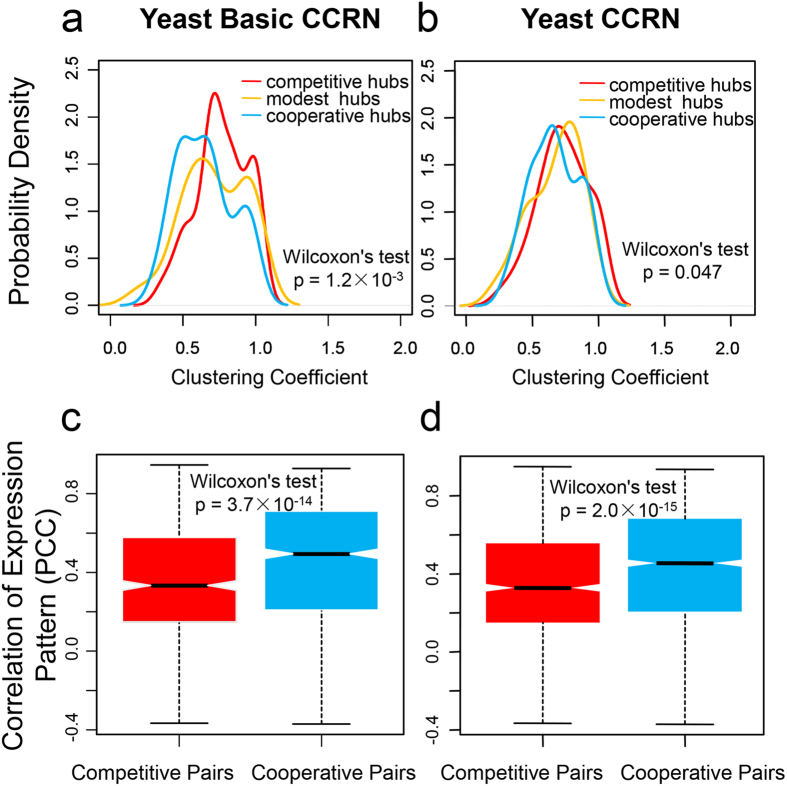
Comparisons of clustering coefficient and PCC distributions in the yeast basic CCRN and the yeast CCRN. (**a**,**b**) The difference in clustering coefficient distributions between competitive hubs and cooperative hubs is estimated using one-tailed Wilcoxon’s test. (**c**,**d**) The correlation of gene expression patterns for a pair of proteins is quantified by PCC. The p-value is estimated from one-tailed Wilcoxon’s test. The black line indicates the median. The range of the box is from the first quartile to the third quartile.

We have ascribed the divergence between the human CCRN and the yeast CCRN to the emergence of the human-specific proteins. However, one may argue that the yeast-specific proteins could also play an important role. Indeed, we could identify 218 yeast-specific proteins (i.e., the proteins containing a yeast-specific Pfam domain) from the yeast CCRN. But this number is much smaller than the number of identified human-specific proteins (1,825). We also constructed the yeast basic CCRN by removing the yeast-specific proteins from the yeast CCRN. This network consists of 593 nodes and 2,136 edges (including 1,182 competitive and 954 cooperative edges). The same analyses as the human basic CCRN were performed for the yeast basic CCRN. First, the proteins that ranked in the top 20% of degree distribution were defined as hubs. In the meanwhile, 39 hubs, 31 hubs and 48 hubs respectively were classified as competitive hubs, modest hubs and cooperative hubs according to the percentage of their connected competitive edges. Then, we compared the distributions of clustering coefficients as well as participation coefficients between competitive and cooperative hubs. As a result, competitive hubs have higher clustering coefficients in the yeast basic CCRN (one-tailed Wilcoxon’s test, p-value = 1.2 × 10^−3^; Fig 6a). This is slightly different from the result in the yeast CCRN (Fig. 6b). By contrast, in terms of participation coefficient, there is no significant difference between competitive and cooperative hubs in the yeast basic CCRN, similar to the result in the yeast CCRN (Supplementary Fig. S12). Finally, we compared the PCC distributions between competitive and cooperative pairs in the yeast basic CCRN. The tendencies are similar in both yeast basic CCRN and yeast CCRN, i.e., competitive pairs have lower PCCs, though the p-value becomes slightly more insignificant for the comparison in the yeast basic CCRN [one-tailed Wilcoxon’s test, p-value = 3.7 × 10^−14^ for the yeast basic CCRN (Fig. 6c) and p-value = 2.0 × 10^−15^ for the yeast CCRN (Fig. 6d)]. Given the small number of the yeast-specific proteins and the limited divergences between the yeast basic CCRN and the yeast CCRN, we conclude that the yeast-specific proteins do not play a major role in the discrepancy between the yeast CCRN and the human CCRN.

## Discussion

In yeast, 1,459 PPIs derive 1,244 competitive pairs. In human, 4,915 PPIs derive 10,463 competitive pairs. Considering that the coverage of current structural PPIs is far from complete, there may actually be a much larger amount of competition in the organisms. In automobile traffic networks, a traffic accident rarely occurs because drivers follow the guidance of traffic lights or polices. However, how such ‘traffic accidents’ are avoided in the organisms remains elusive. We therefore constructed the yeast and human CCRNs to try to explain this issue. CCRNs have advantages over traditional PPI networks. First, CCRNs can more intuitively reflect the competitive or cooperative relationship between two proteins that bind a common protein, whereas all edges are marked uniformly in PPI networks. Second, CCRNs provide a network view of competitive and cooperative relationships, thereby enabling holistic analyses. Last, CCRNs take proteins (rather than interfaces) as nodes, which favors the integrative analyses.

Our current work suffers from two limitations. On the one hand, the coverage of structural PPI data remains insufficient, meaning that the established CCRNs will inevitably contain some biases. On the other hand, the CCRNs are deduced from static PPI networks, which may cause the discordance with *in vivo* relationships between proteins. More specifically, two proteins are considered to be competitive if they interact with a common protein on the same interface. However, if the interactions between the competing proteins and the common protein are weak, they may not cause strong functional implications. Indeed, a number of studies have demonstrated that on the basis of interaction strength, PPIs could be classified into transient or permanent ones^4^,^36^,^37^. If two proteins permanently interact with a common protein on the same interface, they may compete more intensively and should be more strictly regulated *in vivo*. To preliminarily probe this issue, for each interaction (i.e., an edge connecting a competing protein and a common protein) in human PPI network, we estimated its strength by ΔASA, which was defined as the difference of accessible surface areas (ASAs) in the unbound state and bound state. Further, for a competitive pair (i.e., a competitive edge in the human CCRN), the average ΔASA was calculated to measure the strength of competition. Finally, we ranked the competitive edges according to their strengths and gradually removed the weak ones. The significance for which the human-specific proteins tend to be regulated by alternative splicing increases in general, when more and more weak competitive edges are removed (Supplementary Fig. S13). This result indicates that the stronger competition is more likely to be regulated by alternative splicing.

Nevertheless, even two proteins may appear to strongly interact with a common protein *in vitro*, there is no competitive or cooperative relationship *in vivo* if they locate in different cellular components or tissues. With the advance of structural proteomics as well as the rapid growth of available information regarding protein subcellular localization^38^ and tissue specificity, our future efforts will focus on constructing a more complete and dynamic CCRN, which will enable the depiction of cellular circumstance at a higher resolution.

## Concluding remarks

In this work, we construct the yeast and human CCRNs based on protein structural interactomes. The analyses of CCRN topology allow us to preliminarily decipher the complicated organization of competitive and cooperative relationships. Due to the existence of human-specific proteins, competitive hubs in the human CCRN, unlike their counterparts in the yeast CCRN, show higher clustering coefficients and form multiple clusters. Detailed analyses further reveal that competitive relationships between proteins could be regulated via various approaches including, but not limited to, differential gene expression and alternative splicing. In summary, the CCRNs could serve as a promising framework wherein one can easily integrate various datasets to perform an in-depth characterization beyond a binary PPI network.

## Methods

### Protein structural interactomes

Yeast and human PPIs with the representative protein-protein complex structures were downloaded from Interactome3D in May 2014^39^. These PPIs were experimentally identified binary interactions and were merged from nine major public databases. For each interaction, protein-protein complex structure was either experimentally determined or modeled by homology modeling. We discarded the self-interactions (homo-oligomers). After these procedures, 1,459 and 4,915 PPIs with associated structural information were obtained for yeast and human, respectively.

### Construction of the yeast and human CCRNs

A protein could have many interaction partners, and the interaction with each partner was depicted by one representative protein-protein complex structure. We noticed that the same residues might have different numberings in different protein-protein complex structures. We unified the numberings according to the amino acid sequence provided in UniProt. Then, residues from different proteins were defined as the interface residues if the distance between their C_β_ atoms (C_α_ for glycine) was less than 7.5 Å (i.e., the C_β_ atom distance cutoff of 7.5 Å was applied)^33^. Furthermore, we examined the interface overlap of any two partners of the same protein by measuring the Jaccard interface similarity. Jaccard interface similarity was calculated by taking the number of residues involved in both of the two interfaces divided by the number of residues involved in either of the two interfaces^14^. If the Jaccard interface similarity was greater than or equal to 0.1, the two partners were defined as a competitive pair connected by a competitive edge in the CCRN; otherwise, the two partners were defined as a cooperative pair connected by a cooperative edge. We used a more stringent threshold 0.1, rather than 0, to exclude the marginal overlap between two interfaces caused by modeling artifacts. It was worth mentioning that if two partners had multiple common interacting proteins, the maximal Jaccard interface similarity would be assigned to the two partners. By the method described above, the yeast and human CCRNs were derived from the original PPI datasets separately.

### Construction of the yeast basic CCRN and the human basic CCRN

Domain information was obtained from Pfam version 27.0 for yeast and human^16^. We found that some domains only existed in the human CCRN. These domains were defined as the human-specific domains, and the proteins that contained the human-specific domains were defined as the human-specific proteins. The rest of the proteins in the human CCRN were classified as the human non-specific proteins. Furthermore, the human basic CCRN was obtained by removing the human-specific proteins from the human CCRN. The yeast basic CCRN was constructed by removing the yeast-specific proteins, following the similar pipeline.

### Hub analyses

We referred to the proteins that ranked in the top 20% of degree distribution as hubs. For each hub, we computed the proportion of competitive edges among all neighboring edges. The hubs were classified according to this proportion into three categories: cooperative hubs (0 to 1/3), modest hubs (1/3 to 2/3) and competitive hubs (2/3 to 1), respectively.

The clustering coefficients for three kinds of hubs were evaluated using the Network Analyzer plug-in provided by Cytoscape^40^. In the CCRNs, the clustering coefficient *c*_*i*_ for protein *i* is defined as:


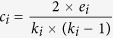


where *k*_*i*_ is the degree of protein *i*, and *e*_*i*_ is the number of edges that connect the partners of protein *i*. The higher clustering coefficient of a protein is, the more tightly its partners link with each other.

We applied the participation coefficient to describe the role of hubs in the modular organization of networks. Note that the participation coefficient requires all nodes in the network be connected^17^. Thus, the largest connected component instead of the whole CCRN was divided into modules by the Markov cluster algorithm (http://micans.org/mcl/). Based on the simulation of random flow across the network, the Markov cluster algorithm can separate the network graph into several non-overlapped densely-connected sub-graphs, i.e., modules. By calculation, each node in the largest connected component of a CCRN could be grouped into one of the modules. To avoid subjective bias, we used the default inflation parameter of 2.0 when implementing the algorithm, which has been widely accepted for typical network topology analysis^41^. After the removal of small modules containing no more than four nodes, the participation coefficient *p*_*i*_ of protein *i* could be calculated as:


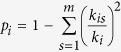


where *k*_*i*_ is the degree of protein *i*, *k*_*is*_ is the number of links of protein *i* in module *s*, and *m* is the number of modules. The participation coefficient of a hub protein ranges from 0 to 1. A node with a participation coefficient close to 0 has all its links within its own module; if the links of a node are uniformly distributed among all the modules, the participation coefficient reaches 1. More details about the participation coefficient can be obtained from literature^17^.

Essential eukaryotic genes were downloaded from Database of Essential Genes (DEG, version 10)^42^. We extracted the essential genes for yeast and human, and mapped them to UniProt accessions by using the ID mapping provided by Protein Information Resource^43^. The proteins encoded by essential genes are essential proteins. Similarly, disease-associated genetic variations were extracted from UniProt^18^ and mapped to the hubs in the human CCRN and the human basic CCRN.

### Regulation analyses

Co-expression data were downloaded from COXPRESdb (Sce.c1-0 for yeast and Hsa.c4-0 for human). In this database, Obayashi *et al.* obtained 2,693 yeast microarray chips and 73,083 human microarray chips from ArrayExpress^44^, and normalized the chips from each organism by the RMA method^45^. Further, for each pair of genes, they calculated the PCC of gene expression patterns based on the normalized expression values^46^. Since COXPRESdb adopted Entrez Gene IDs, we mapped the UniProt accessions to Entrez Gene IDs by using the ID mapping provided by UniProt^18^. Note that a UniProt accession might correspond to multiple Entrez Gene IDs. In this case, we took the average PCC for the gene pairs. In addition, we counted isoform numbers for the human-specific proteins and the human non-specific proteins based on UniProt annotations^18^.

## Additional Information

**How to cite this article**: Li, H. *et al.* Competition-cooperation relationship networks characterize the competition and cooperation between proteins. *Sci. Rep.* **5**, 11619; doi: 10.1038/srep11619 (2015).

## Supplementary Material

Supplementary figures and tables

## Figures and Tables

**Table 1:** Comparison between hub proteins for their associations with essential proteins.

	Yeast CCRN			Human CCRN			Human Basic CCRN		
Com	Mod	Coo	Com	Mod	Coo	Com	Mod	Coo	
Essential protein	11	18	51	58	68	86	17	31	13
Other protein	33	13	38	164	98	105	62	52	24

‘Com’, ‘Mod’ and ‘Coo’ are abbreviations for ‘competitive hub’, ‘modest hub’ and ‘cooperative hub’, respectively. Essential proteins are enriched in cooperative hubs instead of competitive hubs in both yeast and human CCRNs (one-tailed Fisher’s exact test, p-value = 3.6 × 10^−4^ for the yeast CCRN and p-value = 4.4 × 10^−5^ for the human CCRN).

**Table 2:** Comparison between the human-specific proteins and the human non-specific proteins for their associations with alternative splicing.

	Human-specific protein	Human non-specific protein
Isoform number > 1	1099	595
Isoform number = 1	726	477

If a protein has more than 1 isoform, it is regulated by alternative splicing. The results show that the human-specific proteins are enriched with more isoforms (one-tailed Fisher’s exact test, p-value = 7.2 × 10^−3^), indicating that they are more likely to be regulated by alternative splicing compared with the human non-specific proteins.
