# Supplementary material for: Competition-cooperation relationship networks characterize the competition and cooperation between proteins
Source: Sci Rep. 2015 Jun 25;5:11619. doi: 10.1038/srep11619 (PMC4479874; doi:10.1038/srep11619)
Supplement: Supplementary figures and tables [file srep11619-s1.doc]

Competition-cooperation relationship networks characterize the competitive and cooperative relationships between protein interactions

Hong Li, Yuan Zhou^*^ and Ziding Zhang^*^

State Key Laboratory for ArgoBiotechnology, College of Biological Sciences, China Agricultural University, Beijing 100193, China

^*^Corresponding authors (Y.Z.: soontide6825@163.com; Z.Z.: zidingzhang@cau.edu.cn)


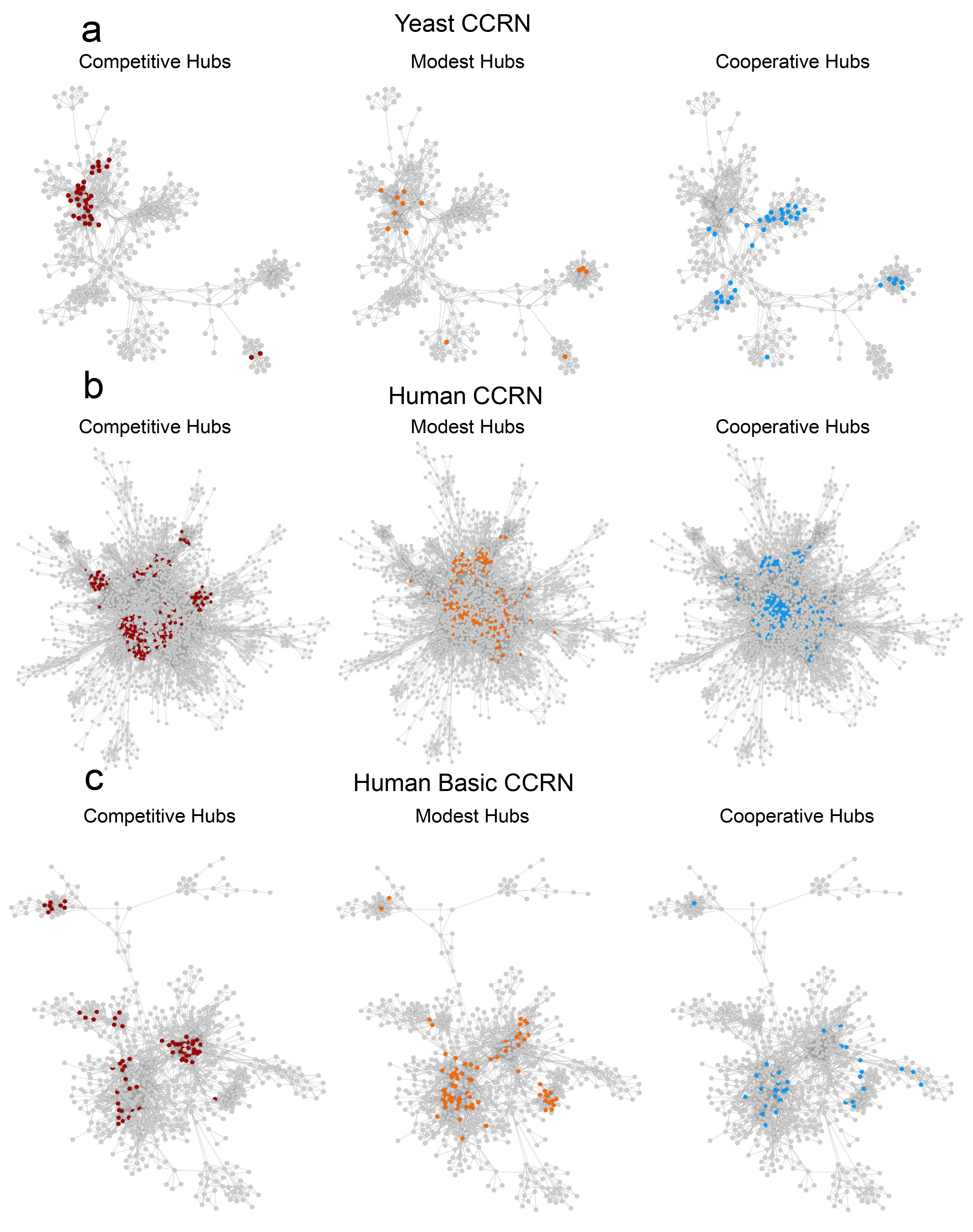


### Figure S1. Distribution of three kinds of hubs.

Competitive hubs, modest hubs and cooperative hubs are highlighted on the largest connected components of the yeast, human CCRN and human basic CCRNs using different colors (red, orange and blue, respectively).


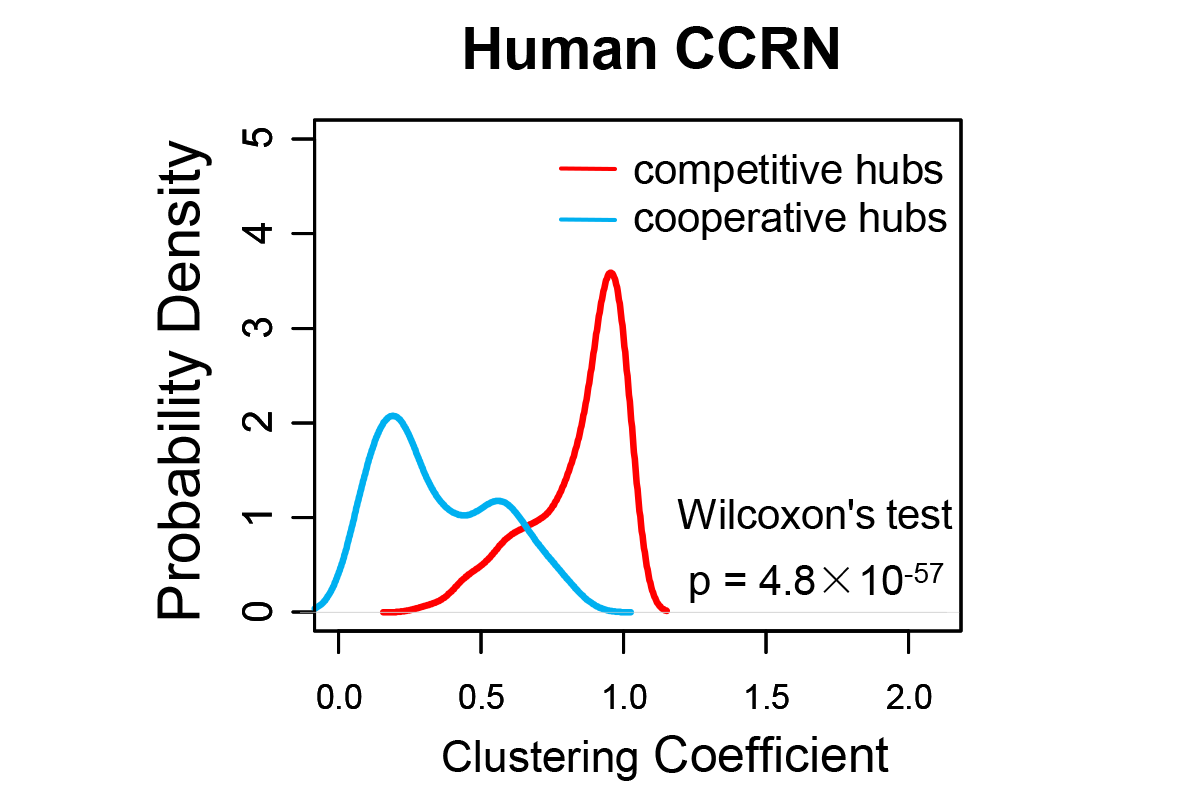


### Figure S2. Comparison of clustering coefficient distributions when only considering competitive edges for competitive hubs and cooperative edges for cooperative hubs.

The difference in clustering coefficient distributions between competitive hubs and cooperative hubs is estimated using the one-tailed Wilcoxon's test.


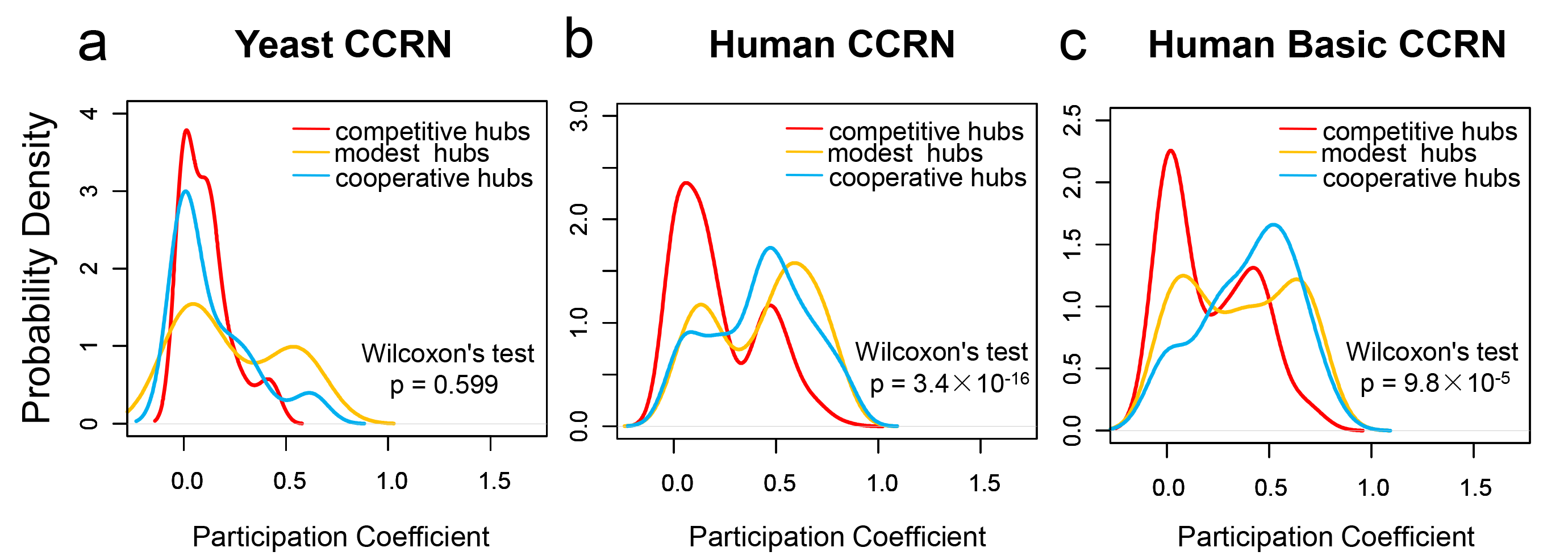


### Figure S3. Comparison of participation coefficient distributions.

The difference in participation coefficient distributions between competitive hubs and cooperative hubs is estimated using the one-tailed Wilcoxon's test.


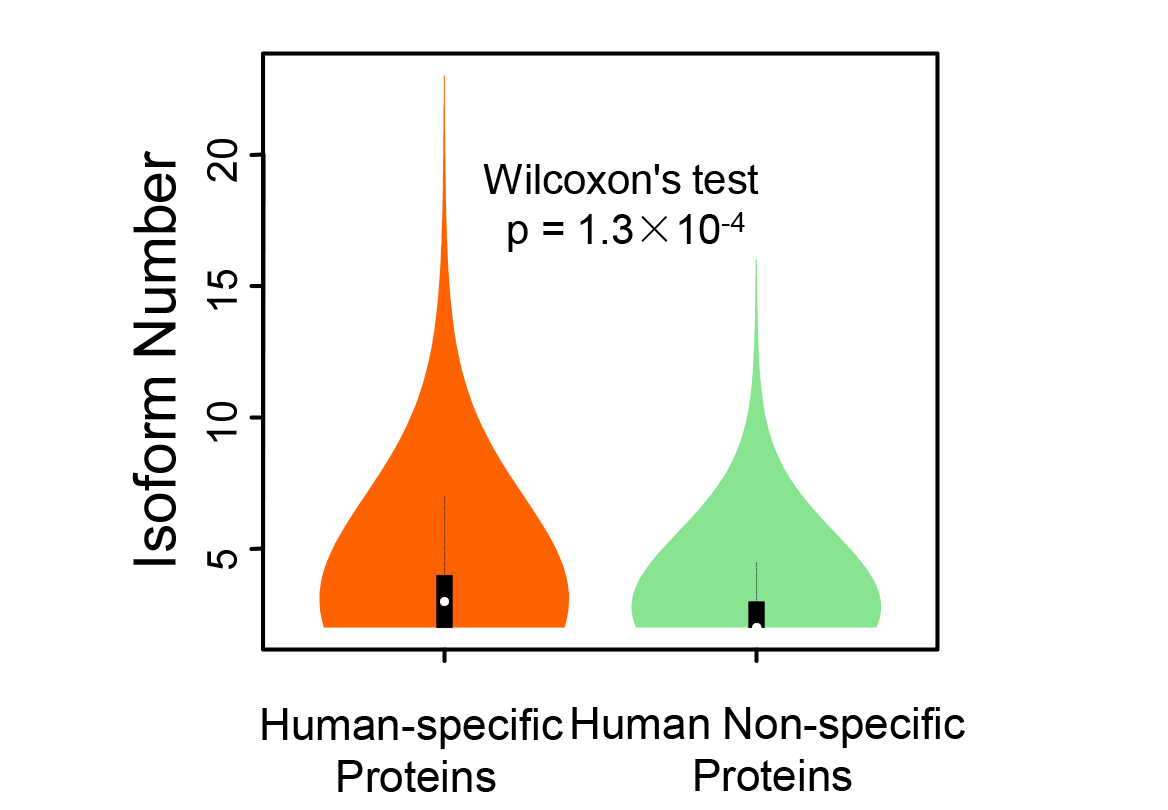


### Figure S4. Comparison of isoform numbers of the human-specific proteins and the human non-specific proteins.

Violin plots represent the isoform number distributions for the human-specific proteins and the human non-specific proteins. The white point indicates the median. The range of the black box is from the first quartile to the third quartile, and the shape depicts probability density.


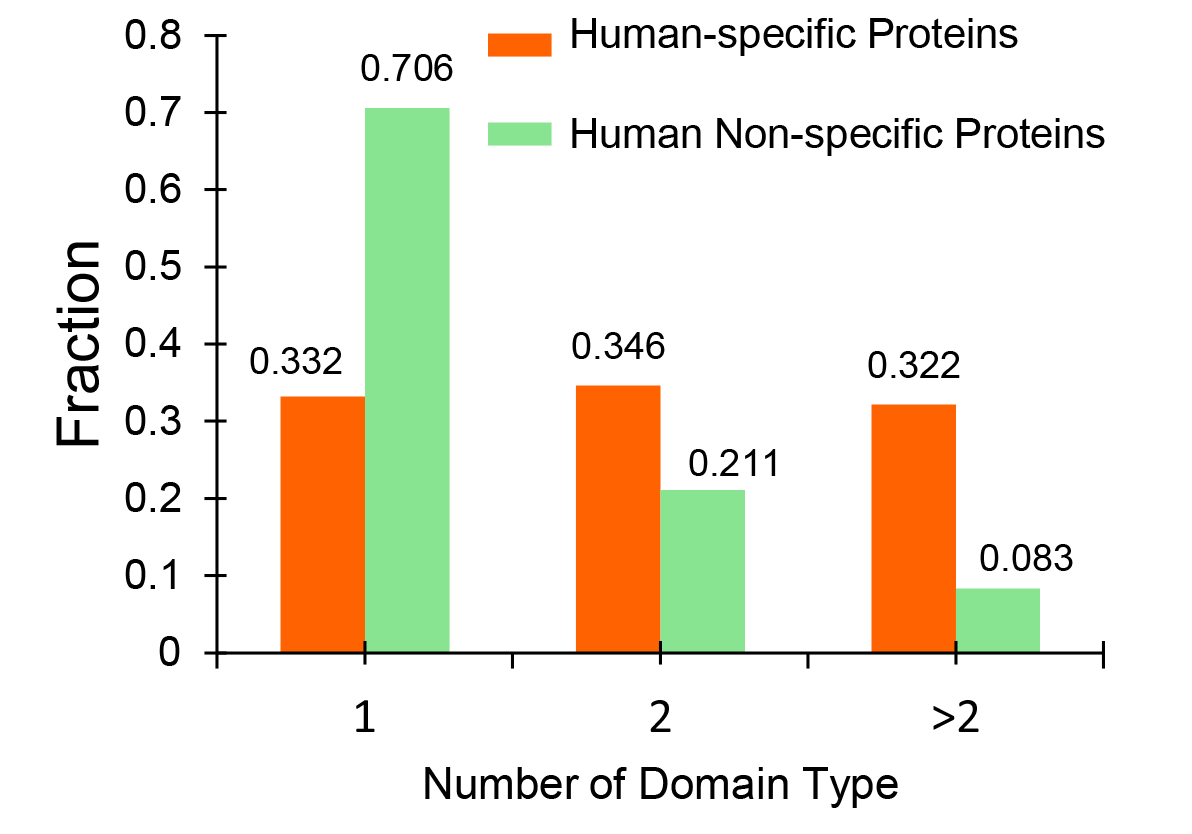


### Figure S5. Comparison of domain type numbers of the human-specific proteins and the human non-specific proteins.

The human-specific proteins and the human non-specific proteins are further classified into the proteins with single domain type, two domain types and multiple (>2) domain types, respectively. Then the percentage of each kind of proteins are calculated.


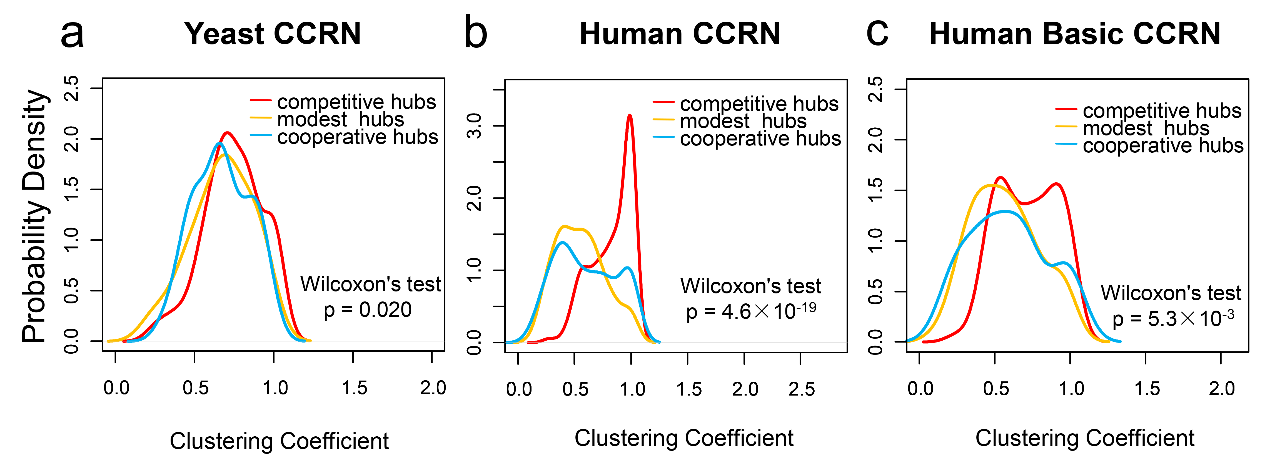


### Figure S6. Comparison of clustering coefficient distributions when a smallest atom distance threshold of 4 Å is used to identify interface residues.

A pair of residues from two different proteins are treated as interface residues if the distance between any two atoms of them is less than 4 Å. The difference in clustering coefficient distributions between competitive hubs and cooperative hubs is estimated using the one-tailed Wilcoxon's test.


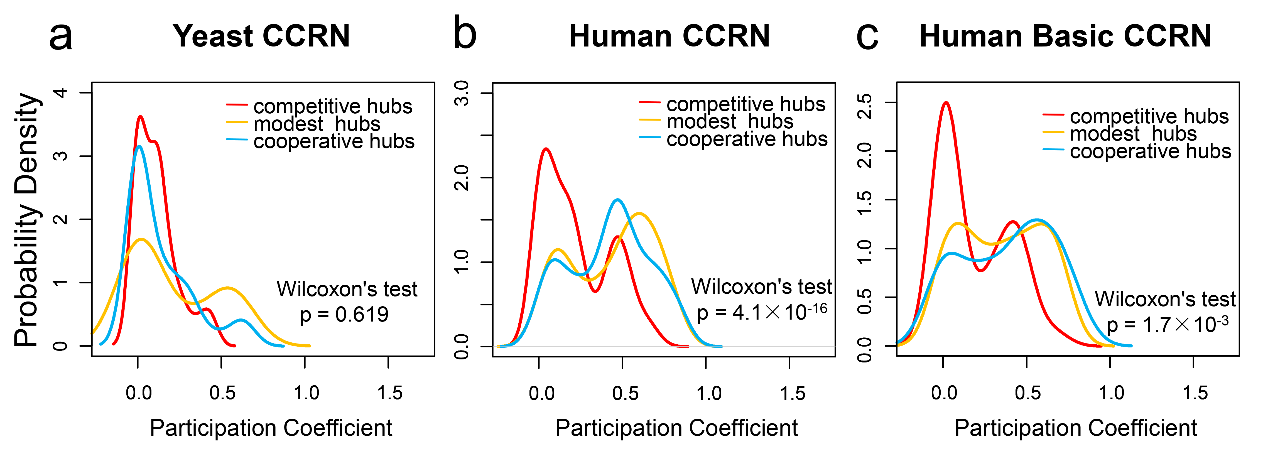


### Figure S7. Comparison of participation coefficient distributions when a smallest atom distance threshold of 4 Å is used to identify interface residues.

A pair of residues from two different proteins are treated as interface residues if the distance between any two atoms of them is less than 4 Å. The difference in participation coefficient distributions between competitive hubs and cooperative hubs is estimated using the one-tailed Wilcoxon's test.


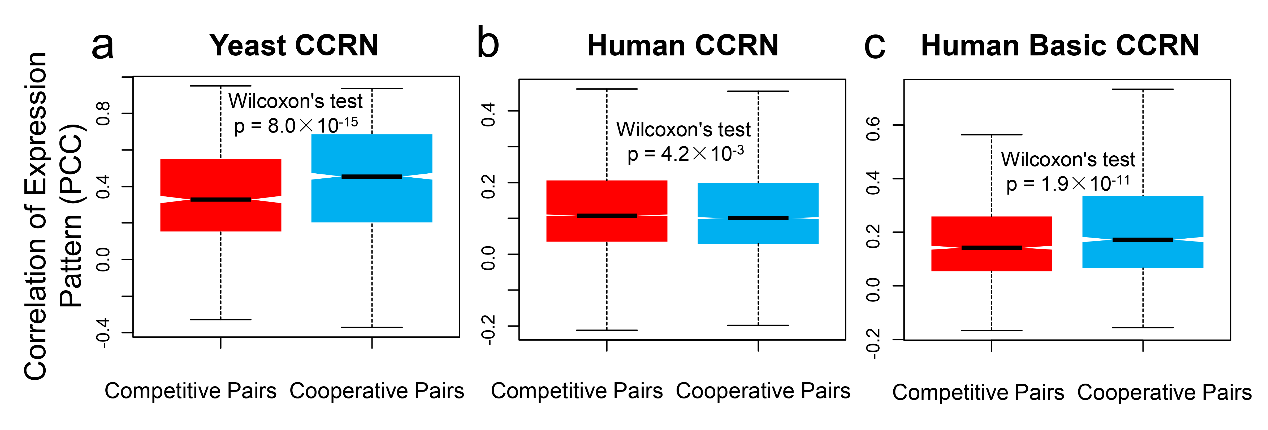


### Figure S8. Comparison of PCC distributions when a smallest atom distance threshold of 4 Å is used to identify interface residues.

A pair of residues from two different proteins are treated as interface residues if the distance between any two atoms of them is less than 4 Å. The correlations of gene expression pattern for any protein pair are quantified by PCCs. The p-values are estimated from a one-tailed Wilcoxon's test. The black line indicates the median. The range of the box is from the first quartile to the third quartile.


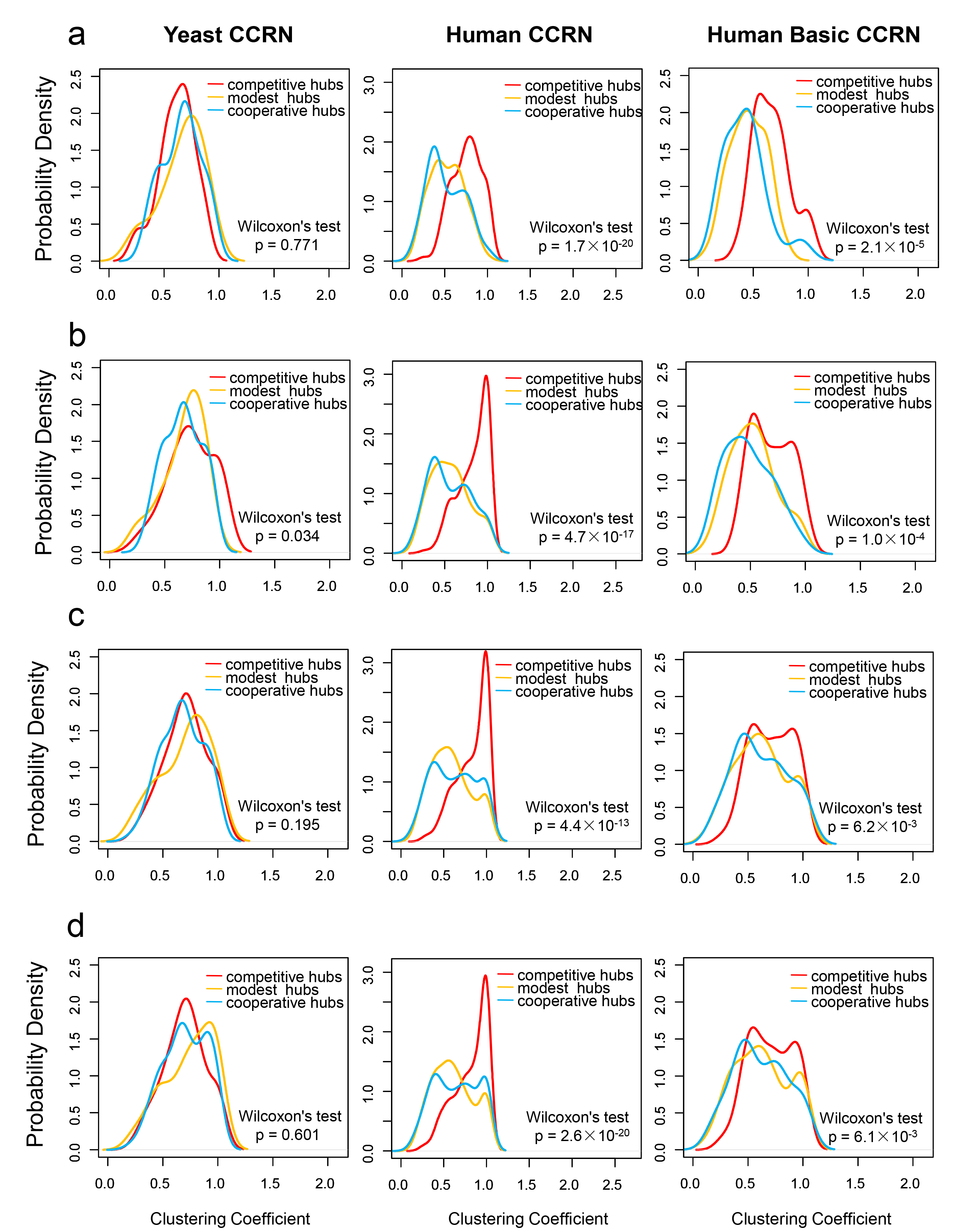


### Figure S9. Comparison of clustering coefficient distributions using different definition of hub proteins.

The difference in clustering coefficient distributions between competitive hubs and cooperative hubs is estimated using the one-tailed Wilcoxon's test. (a) When the proteins that rank in the top 10% of degree distribution are defined as hubs. (b) When the proteins that rank in the top 15% of degree distribution are defined as hubs. (c) When the proteins that rank in the top 25% of degree distribution are defined as hubs. (d) When the proteins that rank in the top 30% of degree distribution are defined as hubs.


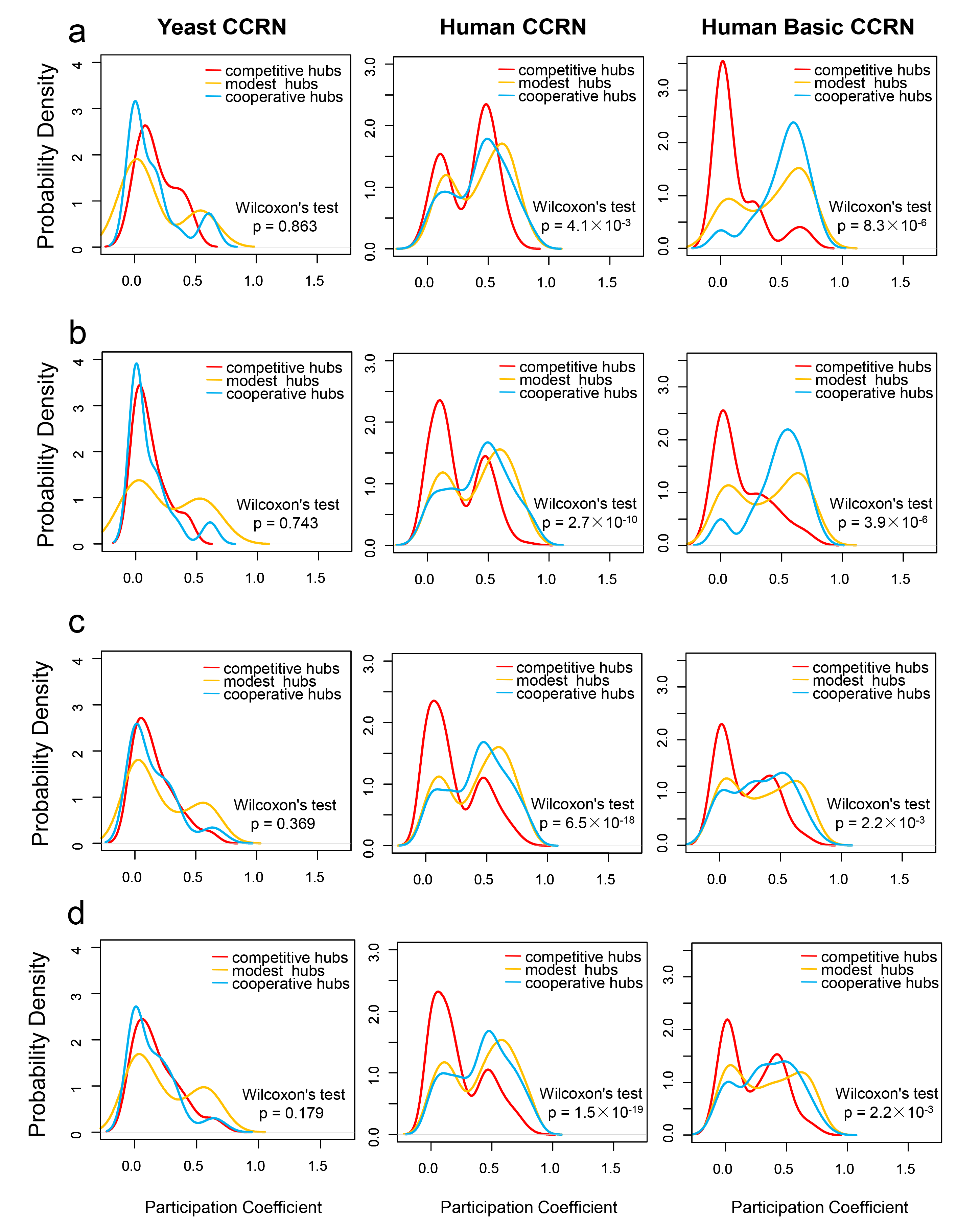


### Figure S10. Comparison of participation coefficient distributions using different definition of hub proteins.

The difference in participation coefficient distributions between competitive hubs and cooperative hubs is estimated using the one-tailed Wilcoxon's test. (a) When the proteins that rank in the top 10% of degree distribution are defined as hubs. (b) When the proteins that rank in the top 15% of degree distribution are defined as hubs. (c) When the proteins that rank in the top 25% of degree distribution are defined as hubs. (d) When the proteins that rank in the top 30% of degree distribution are defined as hubs.


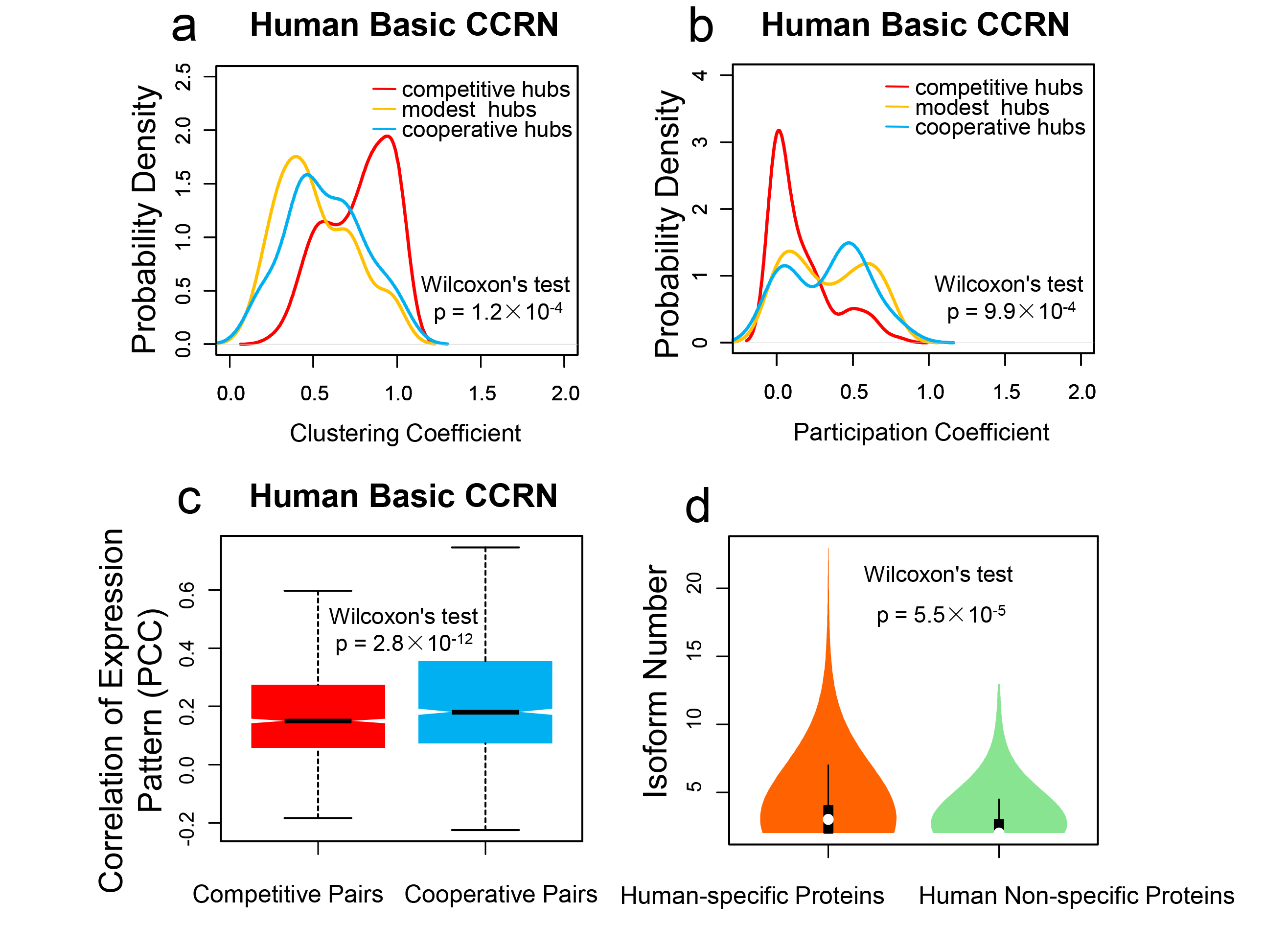


### Figure S11. Analyses of the human basic CCRN where protein family information is employed to identify the human-specific proteins.

Here, instead of protein domain information, protein family information from the PANTHER database was employed to identify the human-specific proteins and the rest of the proteins in the human CCRN were classified as the human non-specific proteins. Furthermore, the human basic CCRN was constructed by removing the human-specific proteins from the human CCRN. (a) The difference of clustering coefficient distributions between competitive hubs and cooperative hubs in the human basic CCRN, estimated by using the one-tailed Wilcoxon's test. (b) The difference of participation coefficient distributions between competitive hubs and cooperative hubs is also estimated by the one-tailed Wilcoxon's test. (c) The correlations of gene expression pattern for any protein pair in the human basic CCRN are quantified by PCCs. The p-value is estimated from a one-tailed Wilcoxon's test. The black line indicates the median. The range of the box is from the first quartile to the third quartile. (d) Violin plots represent the isoform number distributions for the human-specific proteins and the human non-specific proteins. The white point indicates the median. The range of the black box is from the first quartile to the third quartile, and the shape depicts probability density.


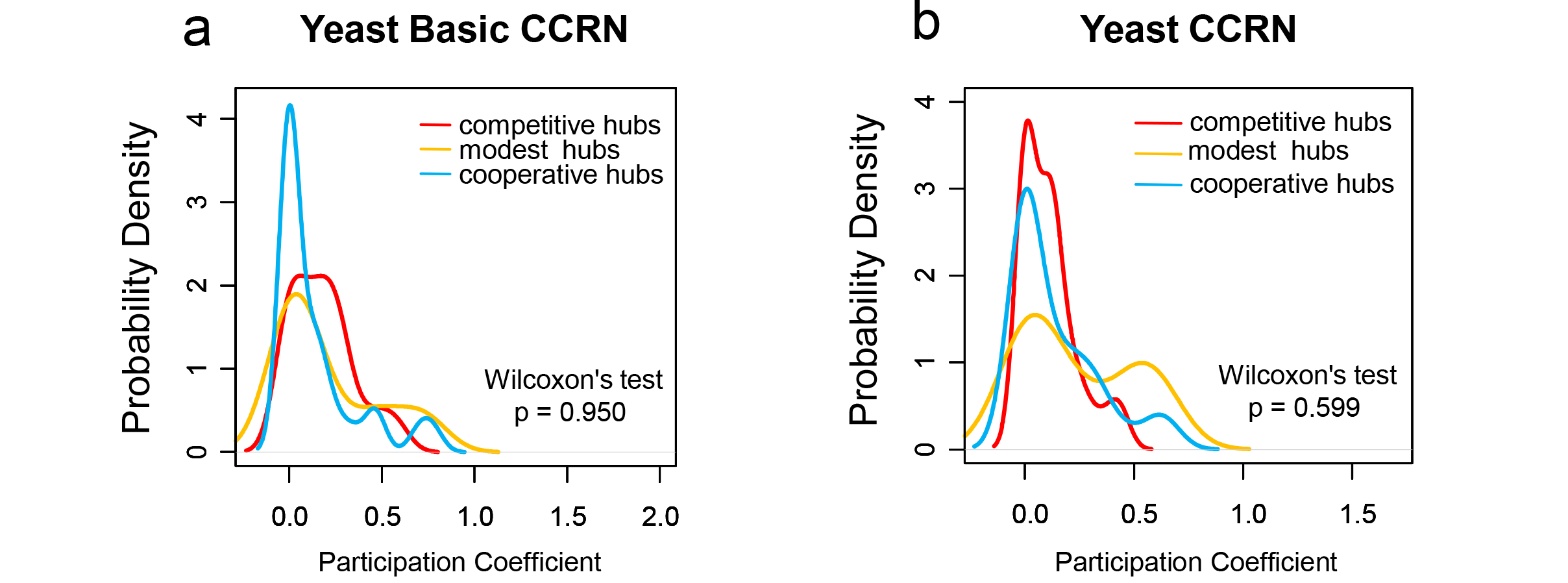


### Figure S12. Comparison of participation coefficient distributions in the yeast CCRN and the yeast basic CCRN.

The difference in participation coefficient distributions between competitive hubs and cooperative hubs is estimated using the one-tailed Wilcoxon's test.


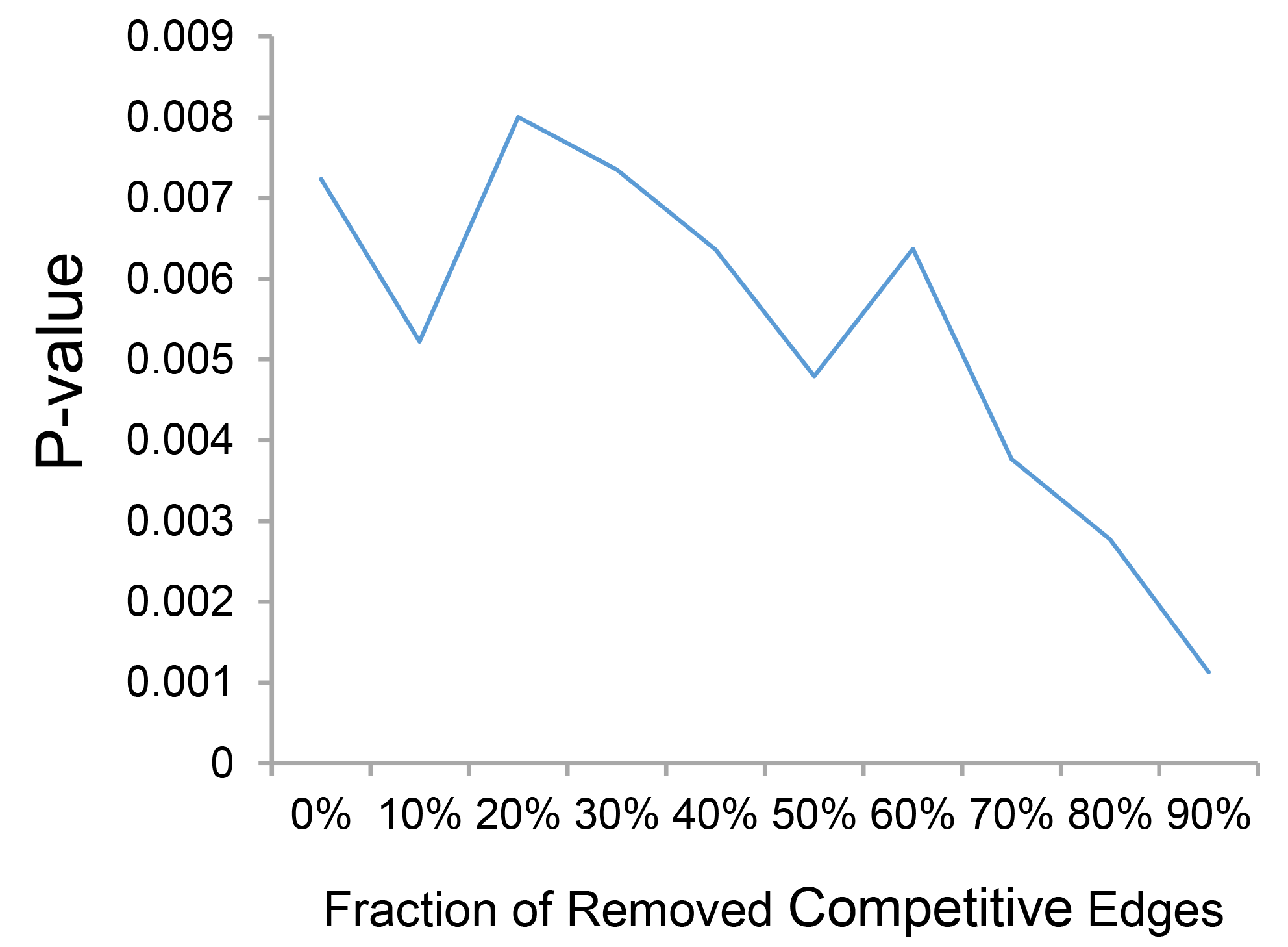


### Figure S13. Enrichment of the human-specific proteins regulated by alternative splicing, varying as the fraction of removed competitive edges increases.

The p-value from Fisher exact test is used to estimate the significance of enrichment. The less the p-value is, the greater possibility for which the human-specific proteins are regulated by alternative splicing is. We gradually removed weak competition edges from the network, and found that the enrichment tendency became largely more significant.

### Table S1. Average degree and the total number of connected components.

|  | Yeast PPIN | Yeast CCRN | Human PPIN | Human CCRN |
| --- | --- | --- | --- | --- |
| Average Degree | 2.564 | 7.796 | 2.813 | 14.851 |
| Connected Component | 219 | 99 | 380 | 191 |

PPIN is the abbreviation of protein-protein interaction network.

### Table S2. Comparison between hub proteins for their associations with essential proteins when an alternative definition was used to assign interface residues.

|  | Yeast CCRN | | |  | Human CCRN | | |  | Human Basic CCRN | | |
| --- | --- | --- | --- | --- | --- | --- | --- | --- | --- | --- | --- |
|  | Com | Mod | Coo |  | Com | Mod | Coo |  | Com | Mod | Coo |
| Essential protein | 9 | 18 | 52 |  | 62 | 63 | 89 |  | 21 | 29 | 11 |
| Other protein | 34 | 11 | 40 |  | 166 | 97 | 103 |  | 64 | 45 | 28 |

Here, a pair of residues from two different proteins are treated as interface residues if the distance between any two atoms of them is less than 4 Å. ‘Com’, ‘Mod’ and ‘Coo’ are abbreviations for ‘competitive hub’, ‘modest hub’ and ‘cooperative hub’, respectively. Essential proteins are enriched in cooperative hubs instead of competitive hubs in both yeast and human CCRNs (one-tailed Fisher's exact test, p-value = 8.2×10^-5^ for the yeast CCRN and p-value = 3.5×10^-5^ for the human CCRN).

### Table S3. Comparison between hub proteins for their associations with essential proteins using different definitions of hub proteins.

|  | Yeast CCRN | | |  | Human CCRN | | |  | Human Basic CCRN | | |
| --- | --- | --- | --- | --- | --- | --- | --- | --- | --- | --- | --- |
|  | Com | Mod | Coo |  | Com | Mod | Coo |  | Com | Mod | Coo |
| The proteins that rank in the top 10% of degree distribution are defined as hubs. | | | | | | | | | | | |
| Essential protein | 1 | 10 | 36 |  | 24 | 40 | 46 |  | 13 | 22 | 5 |
| Other protein | 13 | 7 | 15 |  | 69 | 58 | 52 |  | 27 | 22 | 10 |
| The proteins that rank in the top 15% of degree distribution are defined as hubs. | | | | | | | | | | | |
| Essential protein | 6 | 14 | 45 |  | 41 | 51 | 60 |  | 15 | 28 | 8 |
| Other protein | 25 | 9 | 24 |  | 133 | 75 | 74 |  | 46 | 38 | 14 |
| The proteins that rank in the top 25% of degree distribution are defined as hubs. | | | | | | | | | | | |
| Essential protein | 13 | 23 | 57 |  | 70 | 91 | 107 |  | 22 | 40 | 16 |
| Other protein | 39 | 22 | 51 |  | 190 | 136 | 130 |  | 68 | 64 | 38 |
| The proteins that rank in the top 30% of degree distribution are defined as hubs. | | | | | | | | | | | |
| Essential protein | 15 | 32 | 65 |  | 80 | 108 | 136 |  | 27 | 49 | 20 |
| Other protein | 42 | 30 | 62 |  | 209 | 169 | 167 |  | 77 | 81 | 44 |

‘Com’, ‘Mod’ and ‘Coo’ are abbreviations for ‘competitive hub’, ‘modest hub’ and ‘cooperative hub’, respectively. Essential proteins are enriched in cooperative hubs instead of competitive hubs in both yeast and human CCRNs under any of tested thresholds (10%, 15%, 20%, 25%, 30%) according to one-tailed Fisher's exact test (p-value = 2.3×10^-5^ for the yeast CCRN and p-value = 1.9×10^-3^ for the human CCRN when top 10% degree threshold is applied to define hubs; p-value = 1.9×10^-5^ for the yeast CCRN and p-value = 7.1×10^-5^ for the human CCRN when top 15% degree threshold is applied; p-value = 6.9×10^-4^ for the yeast CCRN and p-value = 1.6×10^-5^ for the human CCRN when top 25% degree threshold is applied; p-value = 1.2×10^-3^ for the yeast CCRN and p-value = 9.4×10^-6^ for the human CCRN when top 30% degree threshold is applied).

### Table S4. Comparison between hub proteins for their associations with essential proteins when protein family information is employed to identify the human-specific proteins.

|  | Human Basic CCRN | | |
| --- | --- | --- | --- |
|  | Com | Mod | Coo |
| Essential protein | 21 | 29 | 11 |
| Other protein | 64 | 45 | 28 |

‘Com’, ‘Mod’ and ‘Coo’ are abbreviations for ‘competitive hub’, ‘modest hub’ and ‘cooperative hub’, respectively. Essential proteins are not enriched in cooperative hubs in the human basic CCRN (one-tailed Fisher's exact test, p-value =0.030).

### Table S5. Comparison between the human-specific proteins and the human non-specific proteins for their associations with alternative splicing when protein family information is employed to identify the human-specific proteins.

|  | Human-specific protein | Human non-specific protein |
| --- | --- | --- |
| Isoform number > 1 | 1137 | 557 |
| Isoform number = 1 | 747 | 456 |

If a protein has more than 1 isoform, it is regulated by alternative splicing. Results show that the human-specific proteins are enriched with more isoforms (one-tailed Fisher's exact test, p-value = 3.0×10^-3^), indicating that they are more likely to be regulated by alternative splicing compared with the human non-specific proteins.
